# Comparing primary care pediatricians’ perceptions of clinics with and without integrated behavioral health

**DOI:** 10.1017/S1463423618000579

**Published:** 2018-08-22

**Authors:** Blake Lancaster, Andrew Cook, Teryn Bruni, Julie Sturza, Jessica Sevecke, Hannah Ham, Rachel Knight, Kathryn Hoffses, Cheryl A. Wickham, Kelly A. Orringer

**Affiliations:** 1 Department of Pediatrics, Michigan Medicine, Ann Arbor, MI, USA; 2 Department of Psychiatry, Geisinger Health System, Danville, PA, USA; 3 Department of Pediatric Psychology and Psychiatry, Nemours/A.I. duPont Hospital for Children, Wilmington, DE, USA

**Keywords:** integrated behavioral health, pediatrics, primary care

## Abstract

*Objective:* The purpose of this study was to investigate primary care pediatrician (PCP) perceptions of prevalence of, time spent in, and satisfaction with behavioral health services across clinics with and without on-site behavioral health providers (BHPs). *Methods:* A cross-sectional survey design was used to examine satisfaction across sites. Data were collected on PCP perceptions of behavioral health services among 60 pediatricians within two academic medical systems. *Results:* PCPs perceived behavioral health issues are prevalent and a time-consuming aspect of medical appointments and preferred to have on-site BHPs over off-site referral sources. Compared to sites without an on-site BHP, sites with on-site BHPs were more satisfied with behavioral health service availability and resources, felt they spent more time addressing medical concerns, and spent less time providing anticipatory guidance. *Discussion:* Study limitations included questions surrounding the validity of survey items to accurately assess PCP perceptions, lack of rigorous experimental design, and reliance on self-report data.

Primary care pediatricians (PCPs) often lack the time and training necessary to meet the increasing demand to effectively address the behavioral health needs of patients within primary care settings (Arndorfer *et al*., 1999; Williams *et al*., 2004; Sheldrick *et al.*, 2011). Several barriers have been noted by PCPs with regards to meeting patient behavioral health needs, including the considerable time commitment required and perceived gaps in behavioral health training (Monson *et al*., 2012). Moreover, PCPs experience lack of financial incentives for the treatment of behavioral health concerns, as the extended time necessary for behavioral health treatment is often not reimbursable within current primary care billing structures (Williams *et al*., 2004; Meadows *et al*., 2011).

A well-researched solution for meeting the behavioral health needs of pediatric patients is integrating behavioral healthcare within primary care settings (Kolko *et al*., 2014; Asarnow *et al*., 2015). Incorporating psychologists as members of the patient-centered medical home improves patient access to essential mental health services and, in turn, improves public health (McDaniel and deGruy, 2014). Physicians reported that integrated behavioral health services led to better communication between physicians and behavioral healthcare providers (Gallo *et al*., 2004; Stancin and Perrin, 2014), a reduction in stigma for patients, and better overall coordination of care (Gallo *et al*., 2004; Torrence *et al*., 2014). The degree of service integration varies from co-treatment and consultation within the medical appointment to co-location, where a behavioral health provider (BHP) is on-site but has a separate schedule to which PCPs can refer patients directly (Heath *et al*., 2013).

The purpose of the current study was to determine if PCP’s perceptions of the frequency of behavioral health issues presented in their practice match the rate reported in the literature, to determine if the presence of BHPs in clinic altered pediatrician perception of time spent addressing behavioral health issues, and to evaluate pediatrician satisfaction regarding the collaboration with on-site versus off-site BHPs.

## Method

### Participants and setting

Participants were recruited across 20 pediatric primary care clinics within two academic medical systems. The integrated behavioral healthcare program was a relatively new initiative in both healthcare systems; therefore, integrated BHPs had only been placed in about half of the primary care clinics. Integrated behavioral health initiatives had been established for at least two years across all clinics, ranging from two years of implementation to about six years of full implementation. A BHP was considered any licensed mental health provider employed full-time to provide behavioral health services within the same clinic space as primary care services. BHPs could include both doctoral-level licensed psychologists and licensed masters-level social workers.

There were 10 clinics staffed with a BHP. All 10 clinics had an on-site psychologist who saw patients on his/her own schedule, billed with psychology current procedural terminology codes, but shared records and clinic space with the PCPs. This level of integration is considered a co-located model of integrated primary care. This model is considered less integrated than models where the psychologist consults within the context of the medical visit and engages in joint treatment planning, as described in Heath *et al*. (2013). The remaining 10 clinics offered either no on-site mental/behavioral health services or only part-time social work support.

### Procedure

A cross-sectional survey design was used to evaluate PCP perceptions. The survey was developed by the research team and was reviewed by an expert panel that included pediatricians and doctoral-level psychologists with experience working in integrated care. The survey consisted of 37 questions on PCP’s perceived prevalence of and time spent on behavioral health concerns during their visits as well as their satisfaction with patient access to service, patient volume, and pace of day. The questions included Likert scale satisfaction ratings that ranged from 1=very dissatisfied to 7=very satisfied, as well questions requesting PCPs to estimate the percentage of time spent on specific concerns. The final survey was delivered via email to all PCPs (*n*=95) employed across the 20 clinics. Survey responses were anonymously collected over a two-week period using an online surveying platform (Qualtrics Surveying Software, 2015).

### Analysis

Descriptive data were collected on demographics across medical sites and clinic type. Univariate statistics were used to check the distribution of the dependent variables (physician responses) in this study. Bivariate tests (*t*-tests and *χ* ^2^) were used to determine if the two medical center sites differed with regard to prevalence of providers working at locations with an on-site IBH, either patient or provider demographics, or provider reports of satisfaction and time allocation. Similarly, potential differences in demographic variables were evaluated across sites with and without a BHP on-site. A two-way analysis of variance (ANOVA) was conducted to examine potential differences in provider satisfaction and time allocation between the two groups of physicians (with or without an on-site BHP).

## Results

### Sample characteristics

Of the 95 surveys sent out, 60 PCPs across the 20 clinics completed the survey at a return rate of 63.1%. A total of 32 PCPs who completed the survey were from clinic with an on-site BHP and 28 of the respondents came from sites without an on-site BHP. Incomplete surveys were not included in the analysis. Demographic characteristics of the participants (PCPs) and patient characteristics across the 20 clinics surveyed are displayed in Table 1. Distributions of dependent variables were all sufficiently normal. Demographic characteristics did not significantly differ between sites with and without on-site BHPs. The two medical centers differed in terms of prevalence of providers working at locations with an on-site IBH, gender of providers, and patient race/ethnicity; therefore, it was necessary to control for medical center in all models.

**Table 1:** Demographic information for primary care physicians and patients within clinics of practice

	%
Physician demographics (*n*=60 respondents)	
Years of experience	
1–2 years	13.3
3–5 years	10.0
6–10 years	11.7
11–20 years	43.3
20+ years	21.7
Gender	
Female	76.7
Male	20.0
Not disclosed	3.3
Geographic area of practice	
Midwest	58.3
Mid-Atlantic	41.7
Patient demographics (*n*=73 380 unique patients)	
Race	
Caucasian	48.3
African-American	36.5
Asian	2.2
Other	13.0
Language spoken at home	
English	93.9
Other	6.1
Insurance	
Private	50.8
Medicaid	45.8
Other	3.5

### Prevalence, satisfaction, and estimated time spent

PCPs estimated that behavioral health problems presented at some point in approximately 30% of sick visits and was estimated as the primary concern within 23% of visits. Nearly 65% of well visits were estimated to include at least one behavioral health concern. Behavior problems were endorsed as the primary concern in over 35% of visits. PCP ratings indicated that attention-deficit/hyperactivity disorder and behavior management problems represent half of all presenting behavioral health concerns across the 20 clinics sampled. Across the 20 clinics sampled, PCPs estimated spending 36.92% of their time addressing behavioral health and school issues, representing over one-third of their total time spent.

Table 2 details results of the two-way ANOVA models, controlling for medical center across all models. When comparing PCP responses across clinic types (on-site BHP versus no on-site BHP), physicians with on-site BHPs reported significantly higher satisfaction with available resources, reported a greater proportion of time spent on medical problems, and a smaller proportion of time spent on anticipatory guidance. No significant differences were found between clinic types in regards to prevalence of or estimated time spent on behavioral health concerns. PCPs reported similar ratings of satisfaction with pace of day, satisfaction with behavioral training, and satisfaction with patient volume.

**Table 2:** Least-square mean estimates, standard errors, and *P*-values from two-way ANOVA models

	No BHP (*n*=28)	BHP (*n*=32)	*P*
Satisfaction with the pace of the day	4.5 (0.3)	4.1 (0.3)	0.44
Satisfaction with behavioral training	4.2 (0.3)	4.2 (0.3)	0.97
Satisfaction with patient volume	4.8 (0.3)	4.9 (0.3)	0.76
Satisfaction with available resources	4.0 (0.3)	5.0 (0.3)	0.02*
Satisfaction with off-site BHPs	3.3 (0.4)	3.3 (0.3)	0.94
Satisfaction with collaboration with off-site BHPs	3.2 (0.3)	3.2 (0.3)	0.96
Overall satisfaction with on-site BHPs	–	6.34 (1.6)	–
Overall satisfaction with collaboration with on-site BHPs	–	6.44 (1.3)	–
% Time spent on behavior problems	23.8 (2.2)	21.5 (1.9)	0.43
% Time spent on medical problems	37.0 (3.2)	46.1 (2.8)	0.04*
% Time spent on school problems	13.7 (1.3)	15.2 (1.2)	0.42
% Time spent on anticipatory guidance	25.5 (2.1)	17.3 (1.8)	0.006**
% of sick visits that behavioral health concern is presented	32.5 (4.5)	32.1 (3.9)	0.94
% of well visits that behavioral health concern is presented	64.8 (6.1)	64.6 (5.3)	0.89
% of sick visits that behavioral health concern is presented as primary concern	23.3 (3.1)	23.4 (2.7)	0.99
% of well visits that behavioral health concern is presented as primary concern	39.1 (4.2)	31.7 (3.7)	0.20

No BHP=clinics without an on-site behavioral health provider; BHP=clinics with an on-site behavioral health provider. *n*=60; *P<0.05, **P<0.01.

Although most ratings of satisfaction were not significantly different across clinic types (see Table 2), it is important to note that both groups reported similar levels of low satisfaction with availability of and collaboration with off-site BHPs. Average ratings were rated <4 (ie, neither satisfied/dissatisfied), across both clinic types. Sites currently accessing on-site BHPs provided consistently high ratings of overall satisfaction (6.34) with on-site BHPs and high satisfaction with provider collaboration (6.44).

## Discussion

This investigation compares PCPs perceptions and satisfaction with behavioral health service delivery across clinics with and without on-site BHPs. Results indicated that generally PCPs perceived behavioral health concerns as a frequent and time-consuming aspect of clinical care across all clinics. These findings were consistent with previous research that indicates between 9 and 25% of all primary care pediatric patients present with behavioral health concerns as the primary presenting concern (Williams *et al*., 2004; Cooper *et al*., 2006; Meadows *et al*., 2011) and that behavioral health issues occupy a significant portion of primary care appointment time (Cooper *et al*., 2006).

This study provides a unique lens through the eyes of PCPs of how the delivery behavioral health services impacts their day-to-day practice. PCPs reported higher levels of satisfaction with on-site BHPs in regards to their access to resources to treat all presenting behavioral health concerns. Significant differences were found in PCP perceptions of time spent on medical concerns and delivery of anticipatory guidance. PCPs in clinics with on-site BHPs estimated significantly more time spent addressing medical concerns and less time spent on anticipatory guidance. PCPs also reported high levels of satisfaction with on-site behavioral health services and both clinic types reported similarly low levels of satisfaction with collaboration with and availability of off-site BHPs. These findings suggest PCPs perceive that traditional off-site referral practices may not always address patient needs.

The presence of on-site BHPs did not appear to be associated with PCP perceptions of time spent on behavioral health issues during appointments or PCP report of satisfaction with ‘pace of day’. Some previous literature in this area suggests that the introduction of an on-site BHP would improve PCP satisfaction with pace of day by decreasing the time they spent addressing behavioral health issues, thus making the primary clinic more efficient overall (Chaffee, 2008; Cummings *et al*., 2009). In contrast, a study by Cooper *et al*. (2006) found PCPs spend more time discussing behavioral health concerns with patients in clinics with BHPs. Cooper *et al.* hypothesized that increased time spent could be due to increased physician confidence and ability to discuss behavioral health concerns with patients as a result of collaborative care efforts. More research is needed, however, to determine the relationship between pace of day and the presence of on-site behavioral health services. The current study suggests that PCPs do not necessarily perceive less time spent on these issues, even when an on-site BHP is present, lending more evidence to the latter hypothesis that physicians could feel increased confidence in dealing more extensively with such issues when they have BHP support.

Behavioral health has become an increasingly important aspect of primary care. There has been a national movement toward PCPs playing a larger role in the delivery of behavioral health services. Specifically, the American Academy of Pediatrics (AAP) has outlined aspirational guidelines for PCPs and trainees promoting increased participation in preventative and basic behavioral healthcare (AAP, 2009). The line between physical and mental health has become less discrete, and PCPs are under increased pressure to provide behavioral health support to their patients (Ader *et al.*, 2015). Despite the emphasis on behavioral health competency among PCPs, recent surveys cite financial barriers, limited time allotted in appointments, and lack of training as potential challenges PCPs face when delivering behavioral health services [American Academy of Child and Adolescent Psychiatry (AACAP), 2009; Horwitz *et al*., 2015].

Fortunately, integrated care models can provide PCPs with the support they need to more adequately address basic behavioral health concerns (Torrence *et al*., 2014). The AAP and the AACAP emphasizes collaborative partnerships with mental health professionals as essential for the delivery of behavioral health services in the primary care setting (AACAP, 2009; AAP, 2009). Although the dynamic between clinic flow and integrated care is not fully understood, the above findings support the idea that even with integrated care, PCPs will continue (and should continue) to discuss behavioral health concerns routinely with patients; however, they are also able to spend increased time on relevant medical concerns. It is possible, as a result of the increased support and resources provided through integrated care, that PCPs are able to provide both physical and mental health guidance in a more fluid and systematic manner. Integrated models emphasize increased screening and collaborative treatment planning (Ader *et al*., 2015); therefore, PCPs may be allocating time to these tasks rather than searching for referrals and resources to provide anticipatory guidance. The relationship between the presence of an on-site BHP and time spent on anticipatory guidance and relevant medical concerns remains unclear, however, and warrants further study.

As priorities around delivery of behavioral healthcare evolve within primary healthcare, it will be important for BHPs to respond to shifts in practice. BHPs are in a unique position to provide resources, develop prevention and screening programs, and provide behavioral health training to PCPs, which can allow them to address behavioral health concerns more directly. However, co-location also has the potential to provide a ‘leveraging’ effect as described by Cummings *et al*. (2009: 35), which is the idea that integrated behavioral health allows PCPs to focus more on medical issues and less time on concerns they are less equipped to treat. These pursuits need not be mutually exclusive and it is likely that BHPs should be prepared to step into both roles, moving BHPs beyond co-location toward being a more integrated member of the primary care team. More research is needed, however, on the feasibility of highly integrated models in healthcare settings.

### Limitations

Several limitations are noted in this study. First, because no existing survey of PCP satisfaction with BHPs could be found within the literature, the authors developed their survey with only expert panel review of survey questions. Without conducting a formal validation study, potential problems with validity and reliability of the survey instrument must be considered. It should also be noted that due to the use of existing samples, groups were not randomized and sample size was relatively small. Statistical control of systematic group differences had to be conducted to control for non-random differences across groups (ie, patient demographic differences across medical centers). Statistical power may also have been reduced due to the small sample size across the two groups. Additionally, there was variability in the length of time when integrated behavioral health services were implemented across all of the clinics. The duration of established integrated programs was not directly measured or controlled for in this study. There may have been differences in PCP perceptions among clinics who had BHPs longer. Finally, data were only collected on provider perceptions of time spent on behavioral health concerns. For a more accurate assessment of time spent and impact on workflow, more direct observational measures should be used.

PCP perception of integrated models of behavioral health delivery is an important area of further study. The above findings lay the groundwork for future research to further investigate the specific aspects of integrated care that appeal to PCPs and how they perceive it to impact their day-to-day practice and, ultimately, the quality of patient care.
